# Draft Genome Sequence of the First Isolate of Extensively Drug-Resistant *Mycobacterium tuberculosis* in Ireland

**DOI:** 10.1128/genomeA.01002-14

**Published:** 2014-10-09

**Authors:** Emma Roycroft, Micheál Mac Aogáin, Ronan F. O’Toole, Margaret Fitzgibbon, Thomas R. Rogers

**Affiliations:** a Department of Clinical Microbiology, Trinity College Dublin, St. James’s Hospital, Dublin, Ireland; b Irish Mycobacteria Reference Laboratory, Labmed Directorate, St. James’s Hospital, Dublin, Ireland

## Abstract

Extensive drug resistance is an emerging threat to the control of tuberculosis (TB) worldwide, even in countries with low TB incidence. We report the draft whole-genome sequence of the first reported extensively drug-resistant TB (XDR-TB) strain isolated in Ireland (a low-incidence setting) and describe a number of single-nucleotide variations that correlate with its XDR phenotype.

## GENOME ANNOUNCEMENT

Multidrug resistance (MDR) in tuberculosis (TB) threatens the global management of the disease, which is already a leading cause of infectious mortality worldwide, with an estimated 450,000 MDR-TB cases reported in 2012 (1). Approximately 10% of MDR-TB cases (those resistant to rifampin and isoniazid) are further defined as extensively drug resistant (XDR)-TB, due to their resistance to second-line drugs, fluoroquinolones and injectable aminoglycosides (2). Long turnaround times (2 to 4 weeks) for phenotypic drug susceptibility testing (DST) (due to the fastidious nature of the organism) can hamper the appropriate treatment of XDR-TB by delaying access to antibiotic susceptibility data (3). Next-generation sequencing (NGS) can highlight resistance in a timely manner in order to effectively manage treatment and minimize further transmission of resistant strains (4–6).

The first Irish XDR-TB strain was isolated in the Irish Mycobacteria Reference Laboratory (IMRL) in 2005 (IEXDR1) (7, 8). First-line DST was completed within 3 weeks (found to be streptomycin, isoniazid, rifampin, ethambutol, and pyrazinamide resistant), second-line DST within 5 weeks (found to be amikacin, clarithromycin, ciprofloxacin, and rifabutin resistant, as well as capreomycin, clofazimine, and prothionamide susceptible), and the remainder within 14 weeks (found to be *para*-aminosalicylate sodium [PAS] resistant and ethionamide and cycloserine susceptible).

In 2014, NGS was performed to provide further molecular characterization of IEXDR1 (lineage 2 or Beijing strain). Genomic DNA was sequenced using an Illumina MiSeq. Paired-end reads were mapped to the *Mycobacterium tuberculosis* H37Rv reference genome (accession no. AL123456.3) using the Burrows-Wheeler Aligner (9). This yielded a mapped-read depth of 196-fold, covering 97.6% of the H37Rv genome. A consensus sequence was called using the SAMtools mpileup command (10). The IMAGE algorithm was employed to extend contigs and close gaps in the assembly, producing a final draft assembly of 4,340,174 bp, consisting of 109 contigs (11). Single-nucleotide polymorphism (SNP) analysis was performed using Geneious R7 (version 7.1.5; Biomatters); 1,492 SNPs were detected in the assembled genome with respect to the genome of H37Rv, of which 810 were nonsynonymous (depth of coverage, ≥20-fold [average, 276]; variant frequency, ≥95%).

Nonsynonymous mutations were identified in genes Rv0667/*rpoB* (H526Y) and Rv1908c/*katG* (S315T). There is strong correlation between substitutions in *rpoB* (H526Y) and *katG* (S315T) and phenotypic resistance to rifampin and isoniazid, respectively (4, 12). High-confidence SNPs were also found for fluoroquinolone resistance in gene Rv0006 (*gyrA*) (D94A) and aminoglycoside resistance in MTB000019/*rrs* (a1401g) (12). This is consistent with the XDR phenotype of IEXDR1. Other high-confidence mutations found in IEXDR1 for ethambutol (Rv3795/*embB* [M306V]) and streptomycin (Rv0682/*rpsL* [K43R]) correlate with its drug resistance profile (13, 14). SNPs that may confer resistance to pyrazinamide (Rv2043c/*pncA* [G132C]) and PAS (Rv3764c/*thyA* [Q97R]) were also identified, although their specificities and sensitivities are not as well defined (http://www.broadinstitute.org/annotation/genome/mtb_drug_resistance.1/DirectedSequencingHome.html).

Previously described phylogenetically informative polymorphisms (Rv1908c/*katG* [R463L], Rv2629 [D64A], Rv3794/*embA* [C76C, TGC/TGT] and [Q38Q, CAA/CAG], Rv1630/*rpsA* [R212R, CGA/CGC], Rv3919c/*gidB* [E92D], and Rv0486/*mshA* [A187V]) confirm the presence of a Beijing strain (15).

In summary, using NGS, this isolate was confirmed to be XDR-TB in a considerably shorter turnaround time than that for conventional DST. This underlines the potential of NGS in the diagnostic laboratory, especially for MDR- and XDR-TB cases.

### Nucleotide sequence accession number.

This whole-genome sequencing project has been deposited in the European Nucleotide Archive under the accession no. CCJS00000000.
